# PReS-FINAL-2170: Work disability in adult patients with juvenile idiopathic arthritis (JIA)

**DOI:** 10.1186/1546-0096-11-S2-P182

**Published:** 2013-12-05

**Authors:** C Goldenstein-Schainberg, J Gordo, R Krieger, LE Paula, N Aikawa

**Affiliations:** 1 Reumatologia, Faculdade de Medicna, Universidade de Sao Paulo, Sao Paulo, Brazil

## Introduction

Approximately 20% JIA patients enters adulthood with clinically active disease and disabled, therefore work condition may be affected.

## Objectives

To assess the prevalence of work disability among adult patients with JIA regularly attending a tertiary rheumatology center and to determine possible associated risk factors.

## Methods

This was a cross-sectional study that enrolled 43 JIA patients according to 2004 revised ILAR criteria. A questionnaire was developed in order to evaluate working status and labor activity: occupation, current/previous work, employment status and withdrawal rate were actively searched. Demographic data, JIA characteristics, clinical activity (DAS28>2.6), therapeutic intervention, comorbidities, physical activity, sedentarism (WHO definitions), functional class (1991 ACR criteria), HAQ and SF-36 were recorded. The prevalence of work disability was calculated using 95% confidence interval, and compared to all parameters; qualitative variables were analyzed using tests of association (chi-square test) and quantitative variables by Mann-Whitney or student test.

## Results

Patients' mean age was 29+7.4 yrs (range 19-41) with mean JIA duration = 17.2+12.3 yrs (range 3-33); 63% were males and 37% females. JIA subtypes were 64% polyarticular, 11% oligoarticular, 9% systemic, 9% ERA, 2% extended oligoarticular, 2% psoriatic arthritis; 7% had uveitis. Serum RF was positive in 21% and ANA in 21%. The majority (72%, n = 31) of JIA patients were employed, whereas 28% (n = 12) were currently not working. In the latter group, 83% (10/12) were retired due to JIA related disability. Further analysis comparing those currently working vs. Those not working revealed similar age (25,3 yrs vs.29,5 yrs, p = 0,09). Although not significantly, most patients currently working had Poly onset JIA (22 vs. 6 p = 0,37), higher frequencies of good education level >12 yrs of school (31 vs.9, p = 0,38), functional class I (p = 0,96), practiced regular physical activity (9 vs. 0, p = 0,89), were singles (26 vs. 8, p = 0,15). Both groups had comparable HAQ and DAS 28 scores (0,62 vs. 0.59, p = 0,47 and 2,51 vs.2,07, p = 0,64) and similar arthroplasty rate (8 vs. 4, p = 0,427). Frequencies of hypertension (3 vs.1, p = 0,999), dyslipidemia (1 vs. 1, p = 0,125), diabetes (1 vs. 0 p = 0,999), depression (1 vs. 0, p = 0,999) and smokers (3 vs. 1, p = 0,99) were alike in both groups. Remarkably, employed patients had higher SF 36 mental health component (84.0 vs. 70.42, P = 0.01).

## Conclusion

High prevalence of almost 1/3 work disability and of retirement due to disease related incapacity remain major problems for adult JIA individuals. We also identified worse mental health in employed patients indicating that further research is needed, in addition to intense affirmative disability actions in order to remove possible disabling barriers and to adapt restrictive environments for these patients. Moreover, enhanced strategies and policy for inclusion of JIA patients in the job market is urged.

## Disclosure of interest

None declared.

